# Characterization of the MurT/GatD complex in *Mycobacterium tuberculosis* towards validating a novel anti-tubercular drug target

**DOI:** 10.1093/jacamr/dlab028

**Published:** 2021-03-16

**Authors:** Arundhati Maitra, Syamasundari Nukala, Rachael Dickman, Liam T Martin, Tulika Munshi, Antima Gupta, Adrian J Shepherd, Kristine B Arnvig, Alethea B Tabor, Nicholas H Keep, Sanjib Bhakta

**Affiliations:** 1 Mycobacteria Research Laboratory, Institute of Structural and Molecular Biology, Department of Biological Sciences, Birkbeck, University of London, Malet Street, London WC1E 7HX, UK; 2 Department of Chemistry, University College London, 20 Gordon Street, London WC1H 0AJ, UK; 3 Research Department of Structural Molecular Biology, Division of Biosciences, University College London, Gower Place, London WC1E 6BT, UK

## Abstract

**Objectives:**

Identification and validation of novel therapeutic targets is imperative to tackle the rise of drug resistance in tuberculosis. An essential Mur ligase-like gene (Rv3712), expected to be involved in cell-wall peptidoglycan (PG) biogenesis and conserved across mycobacteria, including the genetically depleted *Mycobacterium leprae*, was the primary focus of this study.

**Methods:**

Biochemical analysis of Rv3712 was performed using inorganic phosphate release assays. The operon structure was identified using reverse-transcriptase PCR and a transcription/translation fusion vector. *In vivo* mycobacterial protein fragment complementation assays helped generate the interactome.

**Results:**

Rv3712 was found to be an ATPase. Characterization of its operon revealed a mycobacteria-specific promoter driving the co-transcription of Rv3712 and Rv3713. The two gene products were found to interact with each other *in vivo*. Sequence-based functional assignments reveal that Rv3712 and Rv3713 are likely to be the mycobacterial PG precursor-modifying enzymes MurT and GatD, respectively. An *in vivo* network involving Mtb-MurT, regulatory proteins and cell division proteins was also identified.

**Conclusions:**

Understanding the role of the enzyme complex in the context of PG metabolism and cell division, and the implications for antimicrobial resistance and host immune responses will facilitate the design of therapeutics that are targeted specifically to *M. tuberculosis.*

## Introduction

TB is an airborne, infectious disease responsible for 10 million new cases of infection and 1.2 million deaths in 2019 alone.^1^ Despite innovations in diagnostics and improved access to care, the global burden of TB remains substantial and the situation is exacerbated with the spread of drug-resistant TB. The drug discovery pipeline offers woefully limited scope for the future of TB treatment, making it imperative to identify potent, novel chemotherapeutics that are antimycobacterial.

The success of *Mycobacterium tuberculosis* as a pathogen and its innate resistance to many antimicrobial drugs can be attributed, in part, to its unique cell-wall structure composed of covalently linked peptidoglycan (PG), arabinogalactan and mycolic acid.^2^^,^^3^ PG synthesis, remodelling or recycling, could be exploited for novel drug design and discovery.^4^

**Figure 1.**
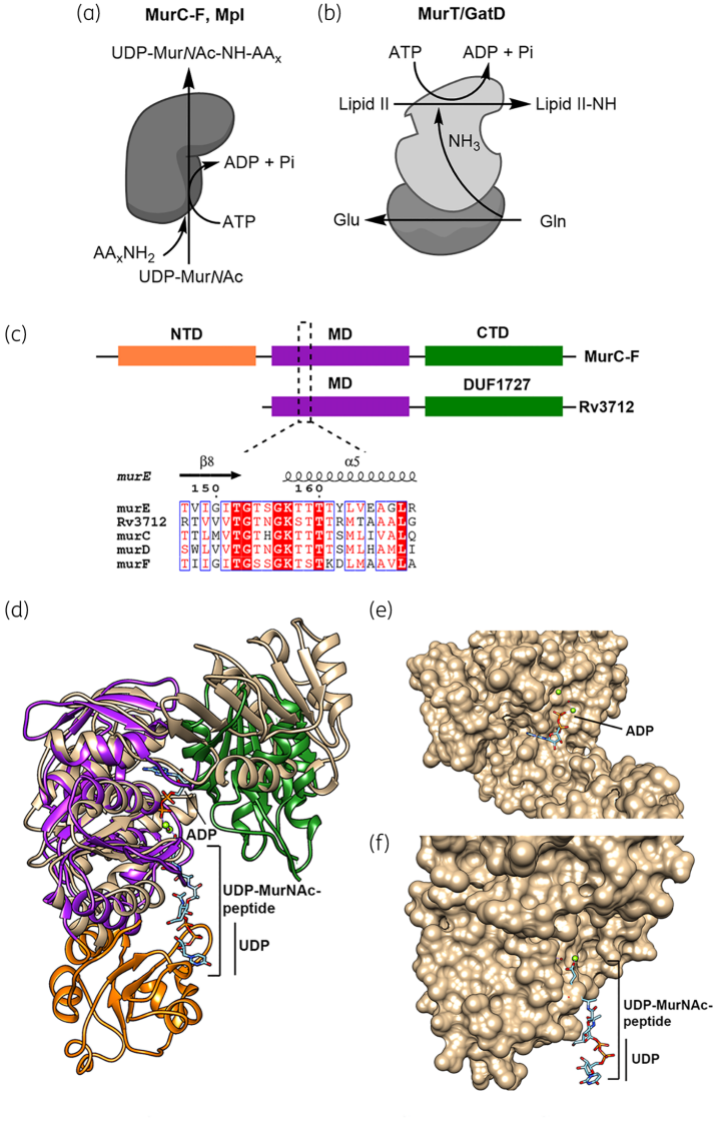
Assignment of function based on sequence similarity and predicted structures. (a) Ligation of amino acid or peptide stem (AA_X_.NH_2_) on to UDP-MurNAc by ATP-dependent Mur ligases and Mpl. (b) Amidation of Lipid II by MurT/GatD complex. (c) Cartoon representation demonstrating the differences in the domain organization of ATP-dependent Mur ligases (MurC-F) versus Rv3712. The three domains in Mur ligases are represented by orange (*N*-terminal domain, NTD), purple (middle domain, MD) and green (*C*-terminal domain, CTD) as are their counterparts in Rv3712. The alignment highlights the sequence identity amongst the proteins in the Walker A motif (P-loop). (d) Superposition of Mtb MurE (PDB: 2XJA) with the predicted structure of Rv3712. The domains of MurE are coloured as above while Rv3712 is in beige. (e) ATP binding region in Rv3712 with ADP position inferred from Mtb MurE. (f) The magnified view of the *N*-terminus of Rv3712 shows that the UDP-binding region is absent.

As the PG-synthesizing Mur ligase enzymes have emerged as potential drug targets,^5–7^ a genome-wide search of *M. tuberculosis* led us to Rv3712—a ‘possible ligase’ expected to be involved in PG metabolism. Rv3712 is essential for the survival of *M. tuberculosis*, it is conserved across all mycobacteria and has survived the genomic decay in *Mycobacterium leprae*, making it an attractive candidate for investigation as a novel drug target.^8–10^ However, its role in PG metabolism was unknown. Based on its predicted ligase activity, Rv3712 could perform one of three PG-associated roles in *M. tuberculosis*: (a) that of an ATP-dependent Mur ligase, involved in *de novo* PG synthesis; (b) that of a murein peptide ligase (Mpl), a PG-recycling enzyme found in Gram-negative bacteria; or (c) that of a MurT, an enzyme which forms a heterodimer with GatD to amidate the peptide stem of Lipid II (Figure 1a and b). The primary aim of our investigation was to identify the role of Rv3712 in cell wall PG metabolism and, through these investigations, to reveal a network of interacting enzymes involved in the pathway, as well as cell division through *in vivo* studies.

## Methods

### Bacterial strains, plasmids and chemicals

*Escherichia coli* strains DH5α (Promega) and BL21(DE3)/pLysS were used for cloning and overexpressing *M. tuberculosis* Rv3712 in the pCDFDuet plasmid. *Mycobacterium smegmatis* mc^2^155 was used with pUAB100 and pUAB200 plasmids for *in vivo* protein–protein interaction studies. pYUB76 constructs were used with both *M. smegmatis* mc^2^155 and *E. coli* for promoter analysis. *M. bovis* BCG was used as the surrogate with pMV261^11^ constructs for the overexpression studies. All restriction endonucleases were purchased from New England Biolabs, primers from Eurofin MWG (Table S1, available as Supplementary data at *JAC* Online). All other media and chemicals were purchased from Sigma–Aldrich unless mentioned otherwise.

### Cloning, expression and purification of wild-type Rv3712 and its mutants

Rv3712 and trigger factor (Rv2462c) were inserted in the multiple cloning site 1 (between EcoRI and HindIII sites) and multiple cloning site 2 (between NdeI and XhoI sites) of pCDFDuet-1 respectively. Site-directed mutants of Rv3712 (G61A and S63A) were generated using the QuikChange Lightning Site-Directed Mutagenesis Kit (Cat#210518) following the manufacturer’s instructions.

The *E. coli* BL21(DE3)pLysS cultures expressing recombinant proteins (wild-type and mutant) were induced with 0.5 mM IPTG and incubated at 18°C overnight (18 h). Post incubation, the cells were harvested, resuspended in lysis buffer (25 mM Tris-HCl pH 8.0, 300 mM NaCl), sonicated and the lysate clarified by centrifugation. The cell lysate was loaded on cobalt resin (HisPur Cobalt Resin, Thermofisher) and Rv3712 eluted at 100 mM imidazole. The protein eluted was directly injected into a pre-equilibrated HiLoad 16/60 Superdex 200 column (GE Healthcare Lifesciences).

### Circular dichroism

Circular dichroism (CD) spectra analysis was carried out on a Jasco J-720 spectropolarimeter. The scans of the spectra were recorded in the UV range from wavelength of 190 to 300 nm with 0.1 mg/mL Rv3712 in 10 mM potassium phosphate buffer with 20 mM NaF. The analysis of CD spectra was carried out using CDtool.^12^

### Assay for ATPase activity of Rv3712

The purified enzyme (Rv3712, 500–2000 ng, mutants—1000 ng, MurC—50 ng) was mixed with substrates (0.1 mM UDP-MurNAc, 1 mM ATP, 1 mM l-Ala) and the final volume made up to 50 μL with buffer (50 mM Tris-HCl pH 8, 5 mM MgCl_2_). The reaction mixture was incubated for 30 min at 37°C. The inorganic phosphate released was detected by the PiColorLock™ Gold Phosphate Detection System (Innova Biosciences) following the manufacturer’s instructions. All reagents and substrates used were individually tested for phosphate contamination. A protein-free reaction as well as an affinity-purified lysate from uninduced *E. coli* was used as a blank to correct the background from the observed results.

### Rv3712-Rv3713 operon analysis

Total RNA extraction was performed as previously described using the FastRNA Pro Blue Kit (MP Bio).^13^ cDNA was synthesized using the Super Script Reverse Transcriptase III kit (Invitrogen). Mock cDNA samples, where Super Script III Reverse Transcriptase was replaced by water served as a negative control to detect genomic DNA (gDNA) contamination. The regions of interest were amplified from the cDNA using Taq DNA polymerase (New England Biolabs). A positive control using *M. tuberculosis* H37Rv genomic DNA was included.

The putative promoter regions, P1 and P2 were cloned into the BamHI site of the transcriptional-translational vector pYUB76. *E. coli* and *M. smegmatis* mc^2^155 cells were transformed followed by blue/white screening on kanamycin (25 mg/L) and X-gal (50 mg/L) plates incubated in the dark at 37°C overnight and for 3 days for each bacterium, respectively. Cells containing pYUB76 with no insert served as the negative control, two positive controls (Rv1409 short/long) of varying strengths containing other mycobacterial promoters were included.^14^

The confirmation of promoter activity was done by measuring β-galactosidase activity in the presence of 2-nitrophenyl-β-d-galactopyranoside (ONPG) as a substrate as described by Miller.^15^

### Protein fragment complementation assay (mycobacterial two-hybrid system)

The bait and prey constructs were generated in pUAB200 and pUAB100 by inserting the gene of interest between the MfeI/ClaI and BamHI/ClaI sites, respectively. The *E. coli* transformants were selected on LB media containing hygromycin (50 mg/L) and kanamycin (30 mg/L) for pUAB100 and pUAB200, respectively. The constructs were confirmed by Sanger sequencing and then electroporated into *M. smegmatis* mc^2^155.

The double transformants were selected on M7H11 supplemented with 0.2% Tween-80, 0.5% glycerol, 0.5% glucose, kanamycin (25 mg/L) and hygromycin (50 mg/L). To detect interaction, five equal-sized colonies were resuspended in 100 μL M7H9. 10 μL of the suspension was streaked on an M7H11 plate containing kanamycin (25 mg/L), hygromycin (50 mg/L) and TMP (12.5 mg/L). Plates without TMP were the growth control. The plates were incubated at 37°C for 5 days. MurC : PknA and Rv3712 : 100 (native pUAB100 vector) were used as positive and negative controls respectively. For quantitative analysis, 100 μL of a 1 : 100 dilution of the above bacterial suspension was added to each well (containing 100 μL of 12.5 mg/L TMP in media) after normalizing the cell density by OD measurements. The plates were incubated for 24 h at 37°C after which 30 μL of 0.01% of freshly prepared resazurin was added to each well. Samples were assayed in triplicate and after 12 h of incubation at 37°C fluorescence was measured at λ_exc_560/λ_emi_590 nm using a fluorimeter (FLUOstar Omega plate reader, BMG Labtech).

### Overexpression of Mur ligases and MurT/GatD in M. bovis BCG and analysis of mutants

The genes of interest were cloned into pMV261 between the EcoRI and ClaI restriction endonuclease sites. *M. bovis* BCG transformants containing pMV261-constructs were maintained in M7H9/M7H10 containing 25 μg/mL of kanamycin. Tb-COLOUR Cold Staining Kit, BDH was used for staining the wild-type and transformant *M. bovis* BCG cells. The antibiotics were tested using a whole-cell phenotypic screen, HT-SPOTi following established protocols.^16^^,^^17^ For each compound, at least three biological replicates were performed.

### Single nucleotide polymorphism (SNP) detection

Information on SNPs present in the clinical isolates was obtained from Genome-wide *Mycobacterium tuberculosis* variation (GMTV) database (https://mtb.dobzhanskycenter.org/cgi-bin/beta/main.py#custom/world).

### Statistical analyses

GraphPad Prism 8.0 was used for all statistical analyses. *t*-tests and two-way ANOVA were used to test the significance of the difference in the means. Alpha was set to 0.05 and the significance was reported as ns=non-significant, *=P < 0.1, **=P < 0.01, ***=P < 0.001, ****=P < 0.0001.

## Results and discussion

### Functional insight into Rv3712 gained from structure

Rv3712 comprises two domains that span residues 56–264 and 303–402 (Figure 1c) as identified by InterPro^18^ and Phyre 2.0.^19^ The first domain is homologous to the central domain of Mur ligases, whereas the second is classified as a domain of unknown function (DUF1727). The central domain of Mur ligases (and Mpl) binds to muramic acid, the peptide moiety and ATP.^5^^,^^6^^,^^20^ ATP binding, in these proteins, is through a canonical glycine-rich mononucleotide binding P-loop (or Walker A motif) with the consensus sequence G-xx-GKT/S.^21^ Rv3712 contains a Walker A motif and shares the highest identity in the ATP binding region when compared with mycobacterial Mur ligases and Mpl from *E. coli* and *Psychrobacter arcticus* (Figures S1a, b and Table S2).

From the sequence and structural alignments (Figure S1a and Figure 1d–f), Rv3712 appears to lack the entire *N*-terminal domain (residues 25–139) found in *M. tuberculosis* MurE (PDB: 2WTZ), which binds to the UDP moiety of UDP-MurNAc (residues between 69–86). As binding of a UDP-linked substrate is critical for the functioning of ATP-dependent Mur ligases (Mur C-F) as well as Mpl, a lack of this domain suggests that Rv3712 is unlikely to be any of these enzymes.

The two-domain structure appears to be a conserved feature of MurT protein, as seen in *Staphylococcus* and *Streptococcus.*^22^^,^^23^ The substrate for MurT is the membrane-bound Lipid II, as opposed to the UDP-MurNAc-peptide substrate of the Mur ligases^24^^,^^25^ and an *N*-terminal domain, in this case, would likely clash with the plasma membrane. Residues for catalytic activity (D349) and ATP hydrolysis (K59, E108) in *Staphylococcus aureus* MurT (*Sa*MurT) are found to be conserved in Rv3712 (Figure S1c).^23^^,^^26^^,^^27^

### Rv3712 is an ATPase

**Figure 2.**
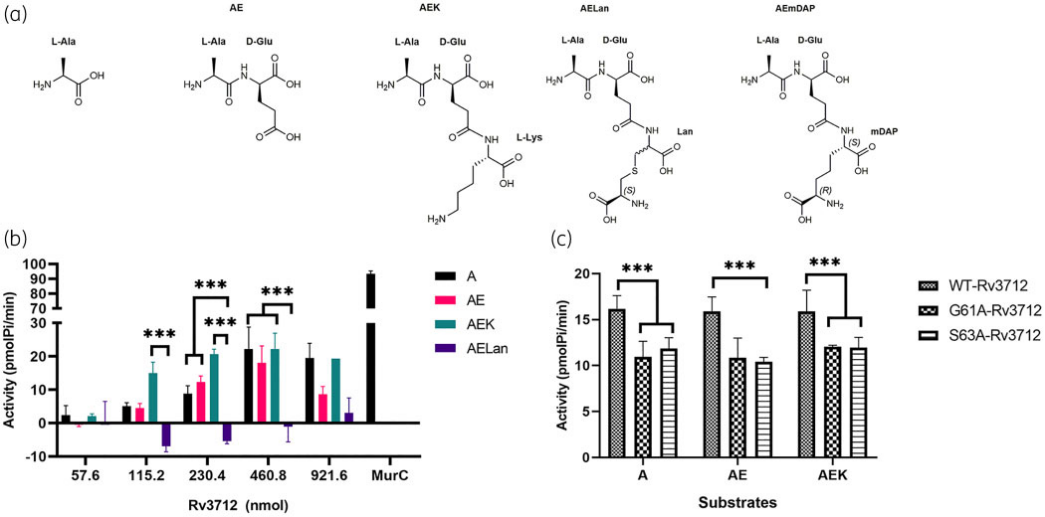
Enzyme activity of Rv3712. (a) Chemical structures of the potential peptide substrates and substrate analogues for Rv3712. (b) ATPase activity of wild-type (WT) Rv3712 in presence of various peptide substrates [l-Ala (A); l-Ala-d-Glu (AE); l-Ala-d-Glu-l-Lys (AEK); l-Ala-d-Glu-Lan (AELan)]. All measurements were obtained in triplicate, the average of which has been plotted. Standard deviations are represented as error bars. *t-*tests were performed to determine statistical significance. (c) ATPase activity of WT-Rv3712 and mutants (460.8 nmol). A decrease in the amount of ATP hydrolysis is observed in the mutants indicating that the residues mutated are critical for this function.

Rv3712, heterologously expressed as a monomer, was found to be folded using CD analyses (Figure S2). Inorganic phosphate release assays with Rv3712 (>90% purity) and substrates of Mpl proteins namely, ATP, UDP-MurNAc, and peptide stems of varying lengths were performed.^28^^,^^29^ In addition to these peptides, we synthesized the mDAP tripeptide analogue l-Ala-γ-d-Glu-Lan (AELan) (Scheme S1). This incorporates lanthionine, which is a thioether-containing bioisostere of mesodiaminopimelic acid (mDAP) (Figure 2a), and is found as an alternative cross-link in the PG of some Gram-negative strains.^30^

Under comparable conditions, Rv3712 exhibits 1/50th of the ATPase activity of mycobacterial Mur ligases (Figure 2b) and that reported of *E. coli* Mpl.^29^ Upon mutating G61 or S63 of the P-loop/Walker A motif (presumed to be crucial for ATPase activity), the inorganic phosphate released by the mutant Rv3712 was reduced, indicating the importance of these residues in enzyme activity (Figure 2c). Comparison of the ATPase activity levels of Rv3712, in the presence of the substrates of Mpl, Mur ligases (MurC-F) and MurT confirmed our hypothesis that it is the mycobacterial MurT.

### Rv3712 forms an operon with Rv3713 under the control of a mycobacteria-specific promoter

**Figure 3.**
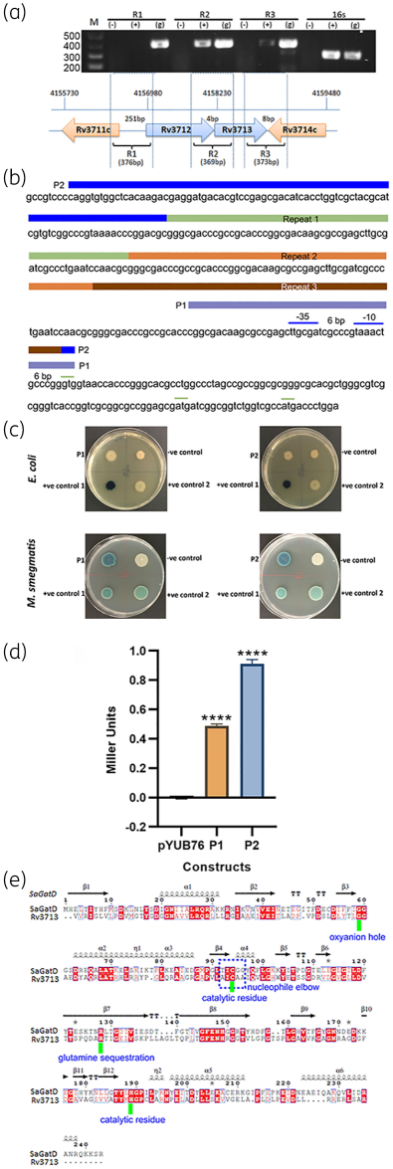
Operon analysis of Rv3712-Rv3713. (a) Amplification of the regions corresponding to R1, R2 and R3 shows that Rv3712 and Rv3713 are co-transcribed and form an operon. (−) negative control containing RNA, (+) cDNA, (g) genomic DNA control. (b) The transcriptional/translational control elements found upstream of Rv3712. The long sequence (P2) and short sequence (P1) selected for the identification of the promoter region are depicted by coloured boxes above the gene sequence. P2 is divided into different colours to depict the repeats observed. The putative −35 and −10 elements have been marked by blue lines above the sequence whereas the annotated start codon and the alternative start codons have been marked by green lines above the sequence. (c) Blue/white colony screening of *E. coli* and *M. smegmatis* mc^2^155 cells containing the putative promoter regions P1 and P2 inserted in pYUB76 containing downstream truncated *lacZ* gene. The negative control was *E. coli*/*M. smegmatis* mc^2^155 with pYUB76 containing no insert (no expression of *lacZ* gene hence colony remains white). Inserts P1 and P2 are not identified in *E. coli* as promoter sequences, however they display strong promoter activity when present in *M. smegmatis* mc^2^155 cells. (d) ONPG assay to identify the promoter strength of the promoter regions P1 and P2 (Miller units represented in the graph provided). The readings are an average of three biological replicates and the standard deviation is represented by the error bars in the graph. (e) Sequence alignment of *Staphylococcus aureus* (*Sa*) GatD and Rv3713. The residues involved in important roles in the dimer complex have been highlighted.

MurT proteins form a heterodimer with glutamine amidotransferase (GatD), and the genes encoding these proteins form an operon in other bacteria.^22^ In the case of *M. tuberculosis*, genome organization suggests that Rv3712 is likely to undergo leaderless transcription and is co-transcribed with Rv3713.^31^ To validate the suspected operon structure, we performed RT-PCR to amplify regions spanning Rv3711c-Rv3712; Rv3712-Rv3713 and Rv3713-Rv3714c, (Figure 3a). The results show a robust signal for Rv3712-Rv3713 (R2), suggesting that these genes are indeed co-transcribed. A faint amplification product is observed for the region R3 (Rv3713-Rv3714c), suggesting that Rv3713 has an extended 3′ untranslated region (UTR), which was confirmed by RNA-seq data obtained from Mycobrowser. *M. tuberculosis* possesses several non-coding RNAs, including long 3′ UTRs, which in many cases overlap with downstream genes and in this case provide antisense RNA to Rv3714c.^32^

A ‘TAAACT’ located 7 base pairs upstream of the transcription start site (TSS) of Rv3712 was identified as the putative −10 promoter element for SigA-dependent promoters (consensus sequence is TANNNT^33^). A putative −35 sequence ‘TTGCGA’ is present 19 bases upstream of the start site of Rv3712 (Figure 3b), but due to the spacing between the two hexamers, it is uncertain if this can function with the identified −10 element. However, studies have shown that it is the sequence of this region and not its position that causes variation in promoter activity.^34^

A 50 bp fragment (P1) and a 270 bp fragment upstream of the Rv3712 TSS (Table S3) were inserted into the promoter probe vector pYUB76, which contains an *N-*truncated *lacZ* gene that lacks the promoter, ribosome binding site (RBS) and start codon. The inserts have the putative promoter elements but no 5′ UTR or Shine-Dalgarno, as native Rv3712 is expressed as a leaderless transcript. *E. coli* DH5α transformed with either reporter construct did not result in blue colonies, when grown on X-gal, suggesting that the reporter fusion was not expressed (Figure 3c). This could be due to a number of differences between *E. coli* and mycobacterial gene expression. Firstly, transcription initiation could be impaired in *E. coli* due to the lack of a canonical −35 element in the correct position relative to the −10 element. Secondly, the leaderless nature of the transcript could affect translation of the *lacZ* gene in *E. coli*, particularly in the context of a GUG start codon.^35^ Thus, the reporter constructs were transformed into *M. smegmatis* mc^2^155, which is a close relative of *M. tuberculosis* with similar transcription/translation machineries. In this case we observed robust expression of *lacZ*, seen as blue colony colour, confirming that the predicted promoter was indeed functional in mycobacteria but not in *E. coli* (Figure 3c).

The longer P2 construct containing the upstream region was found to be around two-fold more active than the shorter P1 construct (Figure 3d) in an ONPG assay. This could be due to activating elements in the promoter upstream region. Within 200 bp upstream of Rv3712 there are three tandem repeats (TR) around 50 nucleotides long. It has been reported that TRs upstream of the −35 site can affect transcription initiation by modifying the binding affinity of regulatory proteins^36^^,^^37^ however, further experimental evidence with appropriate deletions is required to understand whether the TRs upstream of Rv3712 exert any influence on the expression of Rv3712 and Rv3713. In addition to the distinct ‘leaderless’ transcript, Rv3713 lacks a canonical ribosome binding site as opposed to its homologues in *S. aureus* and *Streptococcus pneumoniae.*

Rv3713 is essential, annotated as *cobQ2*, a gene involved in vitamin B_12_ synthesis. However, Rv3713 lacks the ATP-binding site and possesses only two of the three residues of the catalytic triad found in typical CobQ proteins. In contrast, CobQ1 possesses the residues essential to its function and could solely be responsible for CobQ function in vitamin B12 synthesis in *M. tuberculosis*. While Rv3713 aligns poorly with CobQ1 (Figure S3), sequence alignment with *Sa*GatD shows that Rv3713 has conserved residues for glutamine sequestration (R128); the catalytic residues (C94, H189) and G59 residue forming the oxyanion hole to deliver NH_3_ to the mycobacterial MurT (Figure 3e).

Our results suggest that Rv3712 is a Mur-ligase-like gene that forms an operon with a glutamine amidotransferase, Rv3713. This conserved arrangement of an essential Mur-ligase-like gene (*murT* homologue) adjacent to a glutamine amidotransferase along with the sequence features indicate Rv3712/Rv3713 to be the mycobacterial MurT/GatD complex.

### Rv3712 interacts with Rv3713 and forms an interaction network with proteins involved in cell regulation and cell division

**Figure 4.**
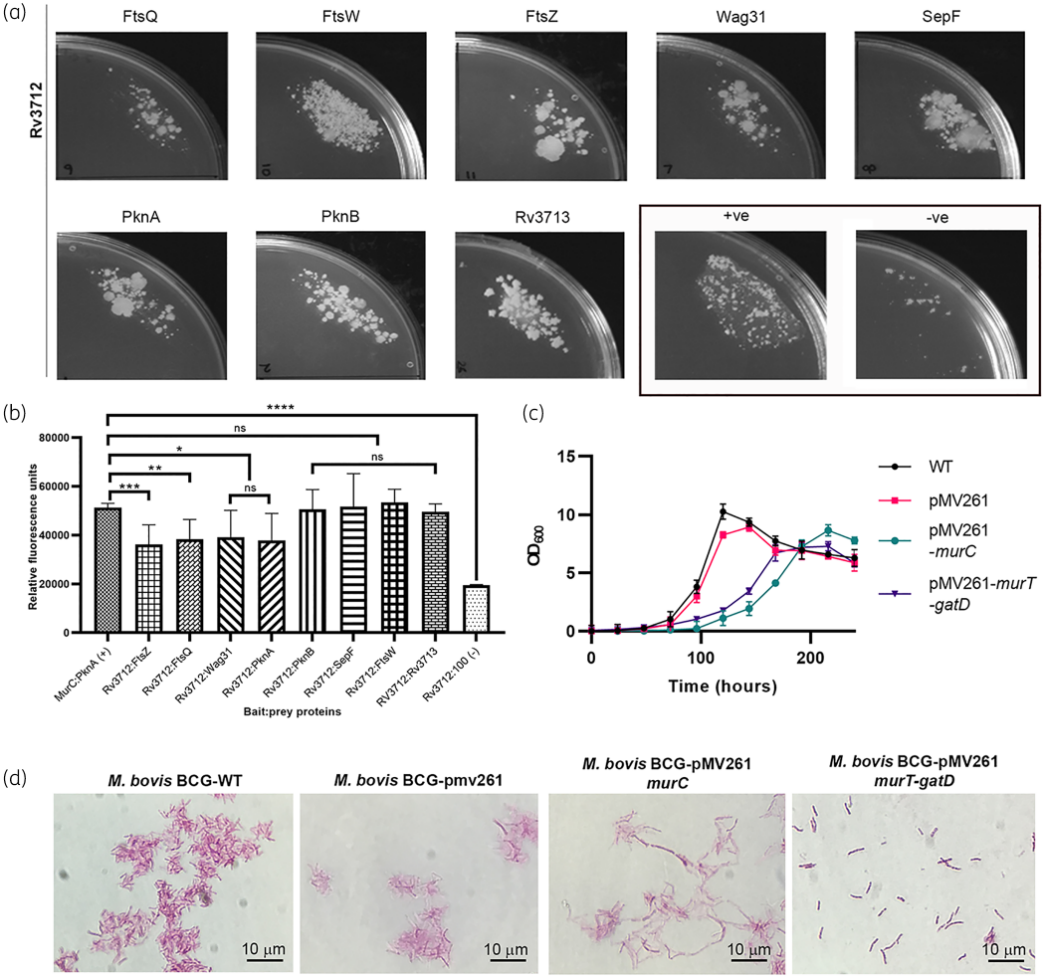
Mycobacterial protein fragment complementation (MPFC) assay and overexpression analysis. (a) MPFC assay of Rv3712 (bait) with prey proteins. Growth of double-transformant *M. smegmatis* mc^2^155 on trimethoprim-containing plates shows interaction between the protein partners. Positive (MurC: PknA) and negative (Rv3712:100) controls were identified from previously published results and selected for this assay. (b) Quantitative results of MPFC assay using resazurin of the proteins pairs as mentioned above. The readings are an average of technical replicates (*n = *3) and the standard deviation of the readings are plotted as error bars. *t-*test was performed for pairs between the experimental controls and the interactions being investigated. (c) Growth curves of *M. bovis* BCG wild type (WT) versus that of those containing the overexpression constructs. Optical density readings were taken in triplicate and averaged for plotting on the graph. (d) Bright-field microscopic observation of acid-fast stained *M. bovis* BCG cells (wild-type and transformed cells) at OD_600_ between 0.8–1.0 under 1000× magnification. Cells overexpressing MurC appear elongated compared with the control wild-type *M. bovis* BCG cells. Cells co-expressing the *murT-gatD* genes appear to be shorter than the control *M. bovis* BCG cells.

As MurT and GatD are known to form a stable heterodimer, a mycobacterial protein fragment complementation (M-PFC) assay^38^ was used to identify the putative protein–protein interaction between Rv3712 and Rv3713 *in vivo*. *M. smegmatis* mc^2^155 cells co-transformed with integrating plasmid (containing Rv3712) and episomal plasmid (containing Rv3713) expressing the respective genes fused to complementary dihydrofolate reductase domains grew on trimethoprim-containing plates suggesting interaction between the two (Figure 4a). The quantitative resazurin assay, supports an interaction between Rv3712 and Rv3713 with a signal of around 50 000 units, where 30 000 units is considered a minimum value for bona fide interactions (Figure 4b). These results strengthen the hypothesis that Rv3712 and Rv3713 are the mycobacterial homologues of MurT and GatD, respectively.

Interactions of MurT with other related/associated proteins were subsequently explored. Putative protein partners included those involved in regulation of cell wall PG synthesis (PknA/B) and cell division (Wag31, SepF, FtsQ/W/Z,) as all of these processes are stringently co-regulated.^39^ M-PFC assays, indicated that MurT interacts with a wide range of proteins (Figure 4). Based on the resazurin assay, MurT seems to have a higher affinity for PknB versus PknA as opposed to Mur ligases (C-F) that exhibited the opposite trend.^7^ The Mur ligases (C-F) were reported not to interact with each other,^7^ a similar trend is observed where Rv3712, a Mur-ligase-like protein, was found not to interact with MurC (Figure S4). Amongst the proteins involved in cell division, MurT was found to interact strongly with SepF and FtsW. The Mur ligases were also seen to interact with these cell division proteins^7^ indicating a complex interactome regulating the processes of cell wall biogenesis and cell division. Understanding the roles of the interacting partners may further reveal the nature of the interaction between the enzymes and MurT. Additionally, co-expressing MurT and GatD and probing the interaction of the heterodimer with the above-mentioned protein partners will provide a definitive picture of the interaction network.

### Overexpression of mycobacterial ATP-dependent MurC and MurT/GatD complex causes phenotypic changes

Overexpression of MurC as well as the MurT/GatD complex in mycobacterial surrogate *M. bovis* BCG show a marked difference in the growth rates of the cells as assessed by OD_600_ measurements (Figure 4c and Table S4). Mechanisms that can slow down growth include interference with the cell cycle and/or cellular respiration. As Mur ligases as well as MurT are ATPases their overexpression could affect the energy pool of the cell. A gross effect on the phenotype when modulating the expression of a single gene indicates a significant role of the gene product in the endogenous pathway making them important drug targets. Overexpression is an evasion mechanism when drugs target a pathway. This can be disadvantageous if the strategy succeeds and leads to resistance. Slowing down metabolism is also a mechanism for increasing tolerance to antibiotics. As TB is always treated by combination therapy, if anti-Mur ligase/MurT inhibitors also demonstrate a similar growth defect, the combination of drugs needs to be tested to confirm bactericidal/sterilizing activity of the suggested therapeutic intervention.

Wild-type *M. bovis* BCG cells are slender, straight or curved rod-shaped bacteria that often appear in clusters. Cells overexpressing MurC displayed an elongated morphology (Figure 4d). In contrast, heterologous co-expression of MurT/GatD (Figure 4d) produced bacilli that were significantly shorter and thicker. Amidation of cell wall PG (performed by MurT/GatD) is known to affect the balance between LD- and DD-crosslinks which in turn is important for the maintenance of the shape of mycobacterial rods.^40^ Thus an increase in the proportion of these links is likely to impact the shape and size of the cells.

The antibiotic susceptibility of the overexpression *M. bovis* BCG strains was tested against front-line anti-TB and cell-wall targeting drugs using HT-SPOTi (Table S5). A change in the susceptibility to antibiotics by altering the expression levels of a single gene indicates that its protein product plays an important role in the endogenous pathway being targeted and/or is involved in the regulation of related genes/pathways. An increase in the MIC of an antibiotic would suggest off-target effects (as none of the enzymes investigated are established targets of the antibiotics used) with implications of cross-resistance that need to be considered during drug design. The MICs of penicillin and vancomycin, drugs targeting the late-stage of PG synthesis, were the most affected in the over-expression strains. Between four- and eight-fold reduction of the MIC was observed against these in MurC, MurE, MurF and Rv3713 overexpression strains as compared with wild-type *M. bovis* BCG. Interestingly, MICs of isoniazid and ethambutol, drugs targeting the mycolic acid component of the cell wall, were also reduced in MurE, MurF and Rv3713 overexpression strains. This indicates that overexpression of these genes regulates the endogenous pathway resulting in cells that are susceptible to attack by cell-wall-targeting agents. Rv3713 is not expected to function as a GatD in the absence of MurT,^24^ so either the overexpressing strain compensates and increases endogenous MurT expression or Rv3713 influences the cell physiology through another feedback mechanism. No difference in the MICs observed in the cases of pyrazinamide, rifampicin, ethionamide and streptomycin indicate that these drugs do not exert off-target effects on these genes, making them novel in the context of drug discovery/design. This reduces the chances of cross-resistance to these drugs as a result of combination therapy with anti-Mur/MurT-GatD-specific drugs.

With antibiotic resistance being so prevalent, it is important that targets being investigated for drug development are not already being targeted, directly or indirectly by current therapy. One way of determining this is to analyse the gene sequence for specific mutations that are enriched in clinical strains of *M. tuberculosis* from patients undergoing treatment. Sequences of the *mur* ligase genes, *murT* and *gatD* from 2800 clinical *M. tuberculosis* isolates show that these genes carry a low level of mutations (Figure S5) that are not selected for, or a result of, targeted selective pressure by the current drugs used in therapy.

### Conclusions

The peptidoglycan of *M. tuberculosis* contains unusual amidated d-glutamic acid in the stem peptide, thereby indicating the presence of MurT/GatD orthologues in the pathogen.^41^ Amidation of PG precursors is important in pathogens as it reduces their susceptibility to lysozyme, other host antimicrobial peptides and antibiotics.^42^^,^^43^

In this study, we have identified Rv3712 as the probable mycobacterial MurT homologue that forms a complex with the downstream GatD homologue, Rv3713. The genes for *murT* and *gatD* always occur in synteny in the organisms in which they have been identified. In *S. aureus* these genes form an operon, which is also the case with Rv3712 and Rv3713. Co-expression of Rv3712-Rv3713 and purification followed by appropriate biochemical assays will provide definitive evidence of their endogenous functions. Rv3713 is annotated in public databases (e.g. Mycobrowser) as cobyric acid synthase *cobQ2*, involved in vitamin B_12_ synthesis. However, Rv3713 lacks several features of CobQ proteins and possesses only the glutaminase domain and two of the residues in the catalytic triad, and falls under the recently described third class of glutamine amidotransferases (GATases) composed solely of GatD proteins.

The M-PFC assay and overexpression experiments revealed a complex network of protein–protein interaction between mycobacterial enzymes involved in cell regulation (PknA, PknB) and cell division (FtsQ-Z, SepF, Wag31).

Novel, essential and druggable targets are urgently needed for anti-TB drug design and discovery initiatives. The mycobacterial cell wall potentially offers targets for both broad-spectrum (in the case of Mur ligases)^44–46^ and narrow-spectrum antibiotics (in the case of MurT/GatD). Further characterization of these targets will benefit the community through increased understanding of the biochemistry leading to the formation of the macromolecular scaffold.

## Supplementary Material

dlab028_Supplementary_Data
