# Supplementary material for: Characterization of the MurT/GatD complex in Mycobacterium tuberculosis towards validating a novel anti-tubercular drug target
Source: JAC Antimicrob Resist. 2021 Mar 16;3(1):dlab028. doi: 10.1093/jacamr/dlab028 (PMC8210147; doi:10.1093/jacamr/dlab028)
Supplement: dlab028_Supplementary_Data [file dlab028_supplementary_data.docx]

**Supplementary data**

**Table S1:** Oligonucleotide primers used in this study. The restriction endonuclease sites have been underlined. Residues in red were added to maintain correct reading frame. Primers with no restriction endonuclease sites have been classified as ‘n/a’.

| **Primers** | **Sequence 5’-3’** | **Restriction sites** |
| --- | --- | --- |
| Rv3712-pCDF-F | TCCGAATTCAGTGGTAACCACCCGGGCA | *EcoRI* |
| Rv3712-pCDF-R | TCCAAGCTTTCAGCCACGACGCGCCAAT | *HindIII* |
| Rv2462c-pCDF-F | CCCCATATGAAGAGCACCGTCGAGCAGT | *NdeI* |
| Rv2462c-pCDF-R | TCACCTCGAGCGTTGTCGCTTCGTCGGA | *XhoI* |
| Rv3712-G61A-F | CGTCACCGGCACCAACGCCAAGTCGACCACCACAC | *n/a* |
| Rv3712-G61A-R | GTGTGGTGGTCGACTTGGCGTTGGTGCCGGTGACG | *n/a* |
| Rv3712-S63A-F | CGTGTGGTGGTCGCCTTGCCGTTGGTG | *n/a* |
| Rv3712-S63A-R | CACCAACGGCAAGGCGACCACCACACG | *n/a* |
| R1-F | GTCCCCAGGTGTGGCTCACAAGACGA | n/a |
| R1-R | CGACCAGACCGCCGATCATCGCTCC | n/a |
| R2-F | GCACTTCGAGAAGACCCGAGTGGTAGC | n/a |
| R2-R | GCGTGTAGAGGTCCAGCGAATCCGGC | n/a |
| R3-F | CGACGGTTTTGATGGCGCGGTTGC | n/a |
| R3-R | GGTGGCCGCCGAGTACGACGGTGATC | n/a |
| 16s-F | AAGAAGCACCGGCCAACTAC | n/a |
| 16s-R | TCGCTCCTCAGCGTCAGTTA | n/a |
| *P2-pYUB76-F | CTTCAAGAATTCCCGGGGATCCCAGGTGTGGCTCACAAGACG | *BamHI* |
| *P2 –pYUB76-R | CGTTGTAAAACGACGGGATCCAGCACCCGGGCAGTTTACG | *BamHI* |
| Rv3712-pUAB200-F | ATTCAATTGGCGTGGTAACCACCCGGGC | *MfeI* |
| Rv3712-pUAB200-R | ATTATCGATGCCACGACGCGCCAATGCTCG | *ClaI* |
| pknA-pUAB100-F | CGCGCGGATCCAATGACCACCCCTTC | *BamHI* |
| pknA-pUAB100R | CGCGCATCGATCTACTGGCCGAACCTC | *ClaI* |
| pknB-pUAB100-F | GCAGTGGCCACCATGAGCCCCCGAGT | *MscI* |
| pknB-pUAB100R | GGATCGATTGGTCATTGCGCTATCTC | *ClaI* |
| FtsQ-pUAB100-F | TGGGGGATCCTATGACGGAACACAACG | *BamHI* |
| FtsQ-pUAB100R | CCGGATCGATCTATTTCACGGTCGGCA | *ClaI* |
| FtsW-pUAB100-F | TTGGGGATCCTGTGCTAACCCGGTTGCT | *BamHI* |
| FtsW-pUAB100R | CCGGATCGATTCACCCGTAACGCTGAC | *ClaI* |
| FtsZ-pUAB100-F | TTCCGGATCCTATGACCCCCCCGCACA | *BamHI* |
| FtsZ-pUAB100R | CCGGATCGATTCAGCGGCGCATGAAG | *ClaI* |
| Rv3713-pUAB100-F | ATTGGATCCAGTGGTGCGGATCGGGCTCG | *BamHI* |
| Rv3713-pUAB200-R | CCGATCGATCTAACGCGCGGATAGCCG | *ClaI* |
| pMV261-murT/gatD-F | ATTGAATTCGTGGTAACCACCCGGGC | *EcoRI* |
| pMV261-murT/gatD-R | CCTATCGATCTAACGCGCGGATAGCCG | *ClaI* |

**Table S2:** Residues involved in ligand interaction in *M. tuberculosis* MurE. The residues in bold were found to abolish ATPase activity of MurE when mutated. The residues marked with an ‘*’ have been mutated in Rv3712 for functional assay.

| Residue | Probable binding | Corresponding residue present in Rv3712 |
| --- | --- | --- |
| L67 | UDP-substrate binding (Uracil) | No |
| A69 | UDP-substrate binding (Uracil) | No |
| Q70 | UDP-substrate binding | No |
| S84 | UDP-substrate binding (Phosphate) | No |
| T85 | UDP-substrate binding (Phosphate) | No |
| T86 | UDP-substrate binding (Phosphate) | No |
| T195 | UDP-substrate binding (Peptide) | No |
| E198 | UDP-substrate binding (Muramic acid) | No |
| S222 | UDP-substrate binding (Peptide) | No |
| R230 | UDP-substrate binding (Muramic acid and peptide) | No |
| H248 | UDP-substrate binding (Peptide) | No |
| G156 | ATP-binding (Phosphate) | Yes* |
| **K157** | **ATP-binding (Phosphate)** | **Yes** |
| T158 | ATP-binding (Phosphate) | No* |
| T159 | ATP-binding (Phosphate) | Yes |
| **E220** | **ATP-binding (Phosphate)** | **Yes** |
| N347 | ATP-binding (Adenine) | Yes |
| R377 | ATP-binding (Phosphate) | No |
| **D392** | **ATP-binding** | **No** |

**Table S3:** The putative promoter regions upstream of Rv3712. The bases in red were not from the WT *M. tuberculosis* H37Rv sequence, but were added to maintain the correct reading frame for translation of the downstream *lacZ* gene. The promoter regions have been underlined.

| Annotation | Length | Sequence |
| --- | --- | --- |
| P1: Rv3712 (short) | 50 bp | Cccggcgacaagcgccgagcttgcgatcgcccgtaaactgcccgggtgaa |
| P2: Rv3712 (long) | 270bp | caggtgtggctcacaagacgaggatgacacgtccgagcgacatcacctggtcgctacgcatcgtgtcggcccgtaaaacccggacgcgggcgacccgccgcacccggcgacaagcgccgagcttgcgatcgccctgaatccaacgcgggcgacccgccgcacccggcgacaagcgccgagcttgcgatcgccctgaatccaacgcgggcgacccgccgcacccggcgacaagcgccgagcttgcgatcgcccgtaaactgcccgggtgct |

**Table S4:** Doubling times of *M. bovis* BCG overexpression strains compared to *M. bovis* BCG wild-type (WT) and the strain containing pMV261 vector.

| Strain | Doubling time (h) |
| --- | --- |
| WT | 16.81 |
| WT pMV261 | 16.50 |
| MurC | 25.17 |
| MurD | 30.89 |
| MurE | 26.49 |
| MurF | 19.75 |
| Rv3712-13 | 24.83 |

**Table S5**: Fold-change in the minimum inhibitory concentrations (MIC) of standard antibiotics against *M. bovis* BCG overexpression strains compared to *M. bovis* BCG strain containing pMV261 vector as assessed by HT-SPOTi assay. ‘-‘ denotes a change in MIC of two-fold change or lower.

| Drugs | Endogenous target | Genes being overexpressed | | | | | | |
| --- | --- | --- | --- | --- | --- | --- | --- | --- |
|  |  | MurC | MurD | MurE | MurF | Rv3712 | Rv3712-13 | Rv3713 |
| Isoniazid | Cell wall synthesis  (Mycolic acid, *inhA*) | - | - | - | - | - | - | 1/4 |
| Ethambutol | Cell wall synthesis  (Mycolic acid) | - | - | 1/4 | 1/4 | - | - | - |
| Pyrazinamide | Membrane disruption | - | - | - | - | - | - | - |
| Rifampicin | Protein synthesis (*rpoB*) | - | - | - | - | - | - | - |
| Streptomycin | Protein synthesis (*rspL, rrs, gidB*) | - | - | - | - | - | - | - |
| Ethionamide | Cell wall synthesis  (Mycolic acid, *inhA*) | - | - | - | - | - | - | - |
| D-cycloserine | Cell wall synthesis (PG, *ddl, alr)* | - | - | - | - | - | - | - |
| Fosfomycin | Cell wall synthesis (PG, *murA*) | - | - | - | - | - | - | - |
| Penicillin | Cell wall synthesis (PG, *PBPs*) | - | - | 1/8 | 1/8 | - | - | - |
| Vancomycin | Cell wall synthesis  (PG, lipid precursors) | 1/4 | - | 1/8 | 1/8 | - | - | 1/4 |


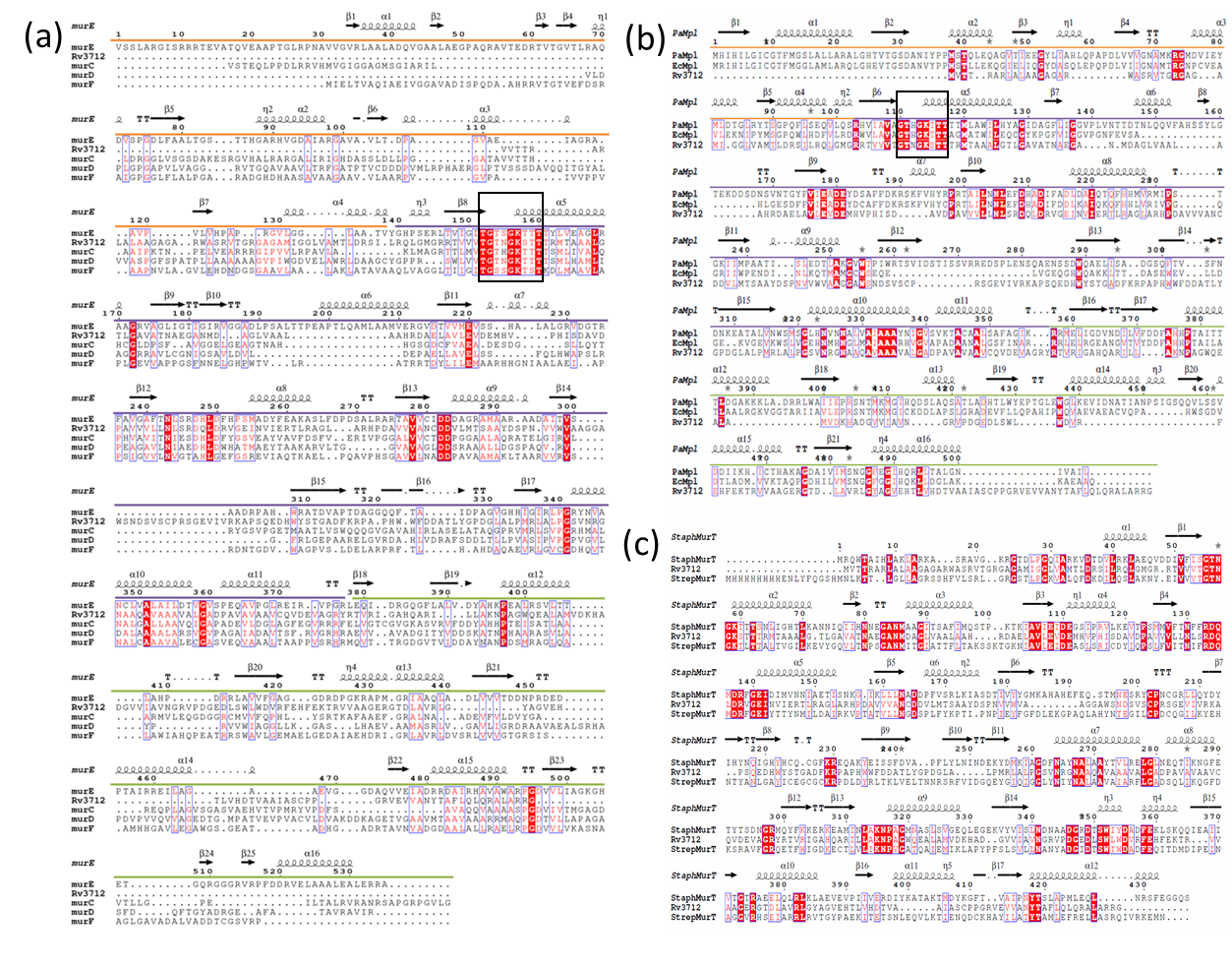
**Figure S1**: (a) Multiple sequence alignment of mycobacterial Mur ligases and Rv3712. The three domains are represented by orange (*N*-terminal), purple (middle) and green (*C*-terminal) bars. The highly truncated *N*-terminal domain of Rv3712 is made apparent by the alignment. (b) Multiple sequence alignment of *Psychrobacter arcticus* (*Pa*) Mpl, *E. coli* (*Ec*) Mpl and Rv3712. The three domains of *Pa*Mpl have been coloured as above (c) Multiple sequence alignment of Rv3712, *Staphylococcus aureus* (Staph) MurT and *Streptococcus pneumoniae* (Strep) MurT.

**Figure S2:** CD spectra of Rv3712 (baseline corrected). The thick, blue line represents the signal wavelength and the thin, black line represents the HT voltage trace.

**
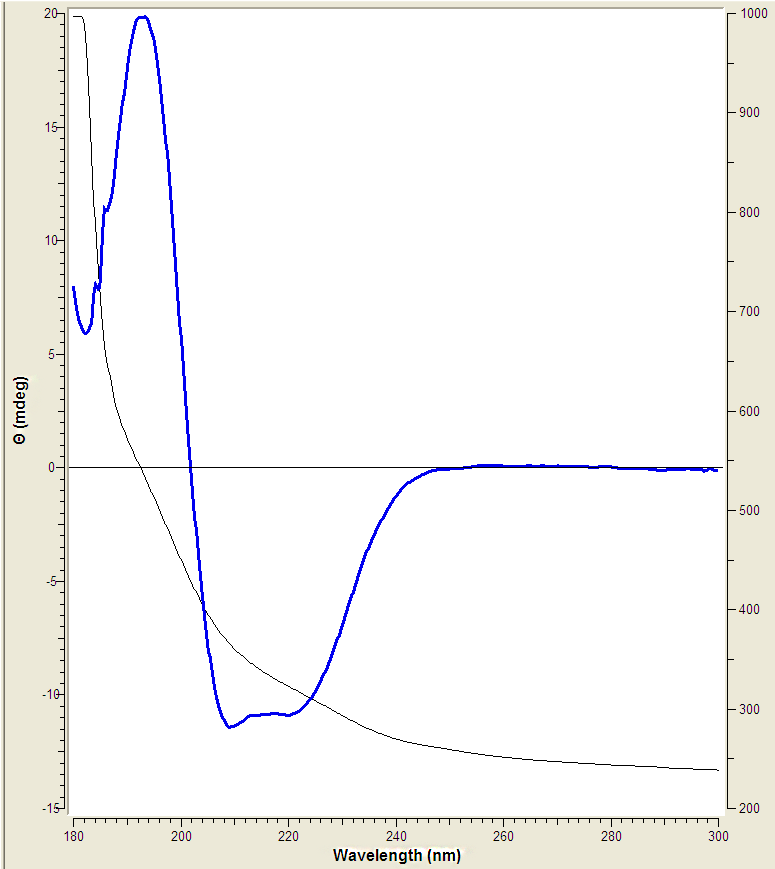
**

**Figure S3**: Sequence alignment of Rv0255c (CobQ1) and Rv3713 (CobQ2).


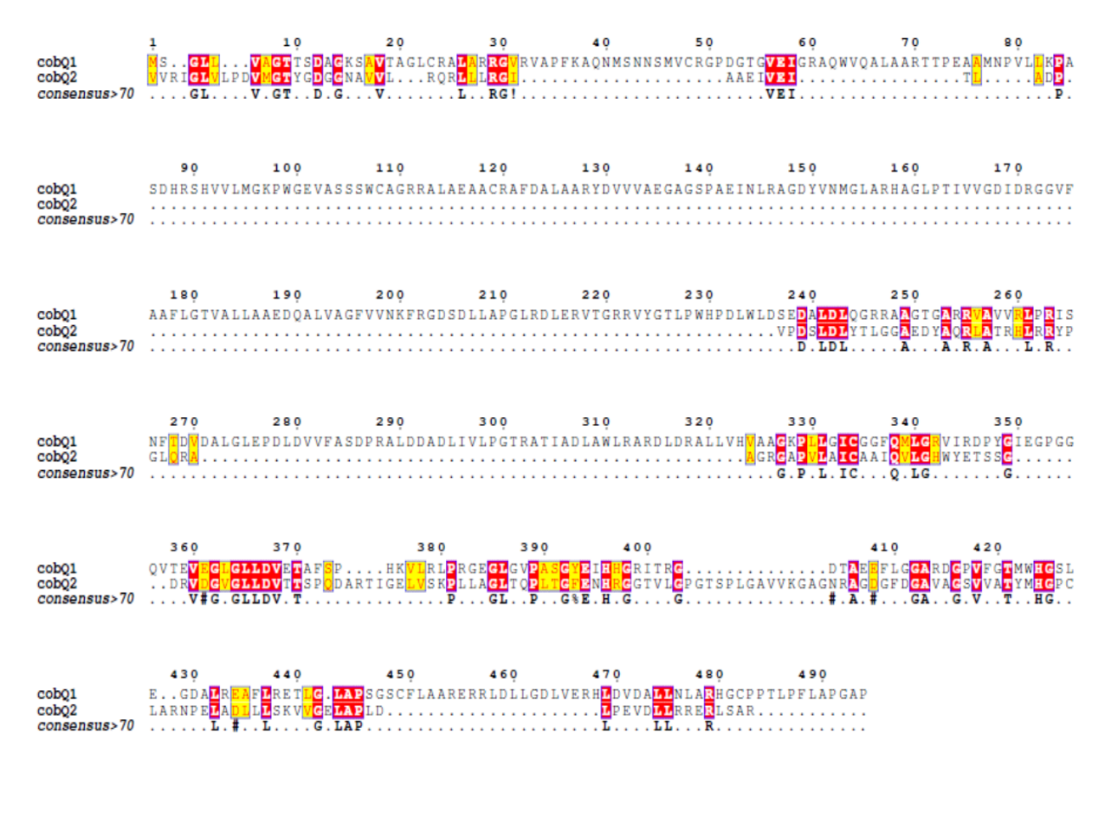


**Figure S4:** MPFC between Rv3712 and MurC alongside positive (MurC:PknA) and negative (MurC:MurF) controls in both the petridish (qualitative) and microtitre plate (quantitative) assay.

**
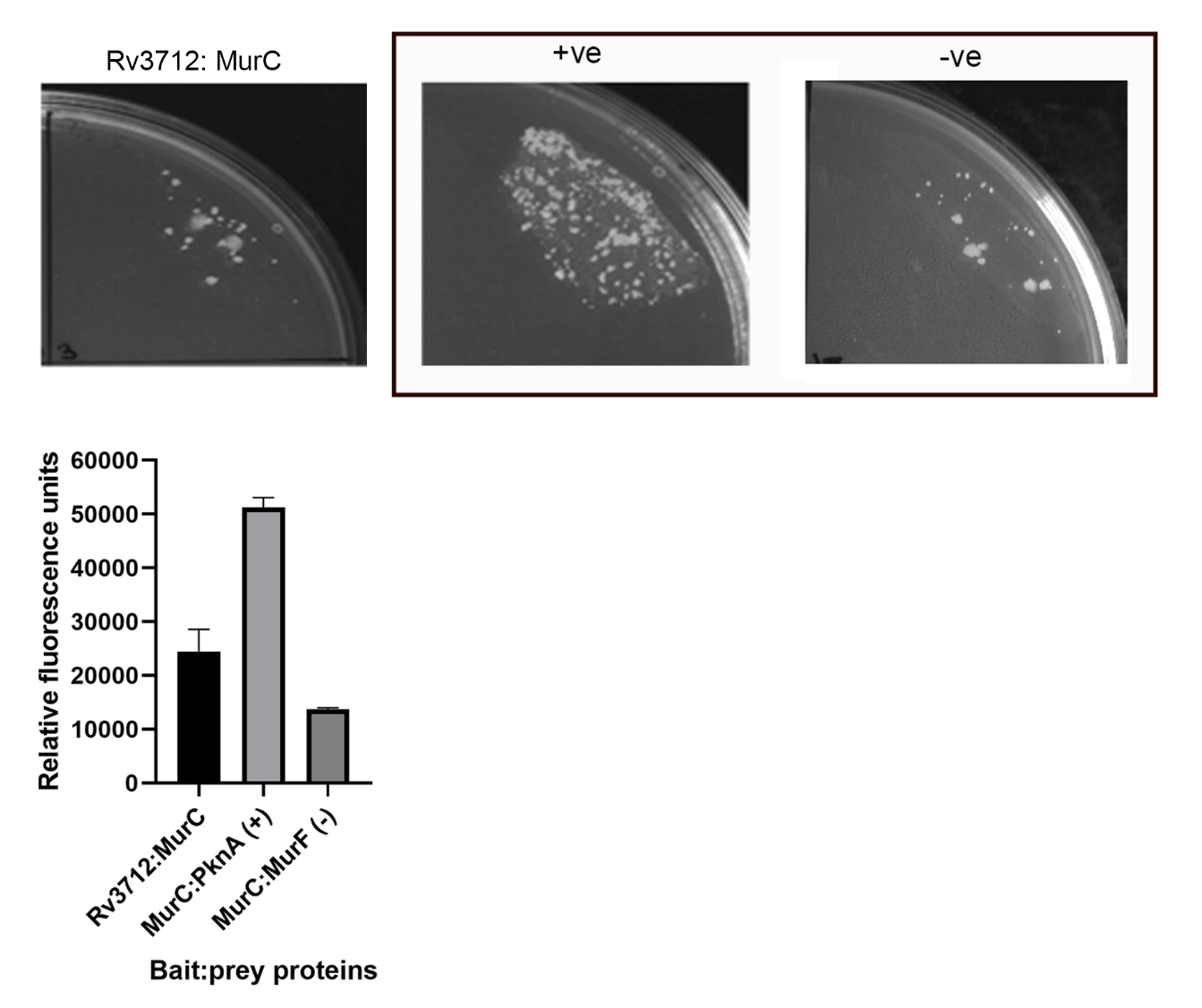
**

**Figure S5: GMTV mutant analyses.** The number of isolates with mutations at a specific codon is mapped in the graphs below. The low frequency of mutations reveal absence of a strong selective pressure such as action of drugs.

**Supplementary Scheme S1:**

**Synthesis of peptides for activity assay of Rv3712**

General Procedures

Anhydrous reactions were conducted under an argon atmosphere, using anhydrous solvents (dried over molecular sieves) purchased from Acros Organics. Resins for peptide synthesis were purchased from Novabiochem, and Fmoc-D-Glu(OAll)-OH was purchased from Bachem. DMF for peptide synthesis, and all solvents used for HPLC, were HPLC grade. All other compounds and solvents for synthesis and NMR were used as purchased from Sigma-Aldrich Co. Ltd. LCMS analysis was conducted on a Waters Acquity UPLC SQD, using a linear gradient of 5-95% acetonitrile in water with 0.1% formic acid over 5 min, using a C8 column and a flow rate of 0.6 mL/min. NMR spectra were recorded on a Bruker Avance-600 spectrometer, with chemical shifts (δ) given in ppm relative to the solvent signal. Carbon signals were assigned from HSQC and HMBC crosspeaks. Abbreviations used in NMR assignment are as follows: d = doublet, t = triplet, q = quartet, m = multiplet. Data processing was carried out using ACD/NMR Processor Academic Edition, version 12.01 (Advanced Chemistry Development Inc.).

Peptides were synthesised by hand using standard Fmoc solid-phase peptide synthesis protocols. The resin was continually agitated throughout coupling, deprotection and cleavage steps by shaking at 480 rpm on an IKA KS130 basic platform shaker. Resins were weighed out in 5 mL PP reaction syringes with frits (Biotage), suspended in DMF (1.5 mL) and left to swell for 30 minutes. After this time the DMF was removed. Removal of the Fmoc protecting group was carried out by sequential treatment of the resin with a 40% piperidine solution in DMF (1.5 mL) for 3 min, followed by a 20% piperidine solution in DMF (1.5 mL) for 10 min. The resin was washed thoroughly between the piperidine solutions and after with 2 and 6 washes of DMF (2 mL) respectively. Coupling of amino acids was carried out by addition of the desired amino acid (5 eq), HBTU (5eq), and DIPEA (10 eq) dissolved in DMF (2 mL), to the resin followed by shaking for 1 hour. The solution was removed, and the coupling reaction was repeated (double coupling) with fresh reagents to ensure saturation. The coupling solution was then removed and the resin washed with DMF (4 x 2 mL). The deprotection-coupling procedure was repeated until amino acids were added, followed by a final Fmoc deprotection. The resin was then washed with DMF (3 x 2 mL), CH_2_Cl_2_ (3 x 2 mL), methanol (2 x 2 mL) and diethyl ether (4 x 2 mL), and dried under vacuum for 1 h. For cleavage, the resin was treated with cleavage solution (TFA:TIPS:water 95:2.5:2.5, 2 x 1.5 mL) and shaken for 30 min. The solution was collected. As these are low molecular weight peptides, ether precipitation was not feasible. Hence the cleavage solution was evaporated under reduced pressure and freeze dried. The residue was redissolved in 10% aqueous formic acid and extracted with chloroform (3 x 2 mL). The aqueous layer was freeze dried. The peptides were redissolved in water and purified using reverse-phase HPLC (Column: Phenomenex onyx monolithic Semi Prep C-18; Dionex 580 HPLC System with PDA-100 photodiode array detector, P580 Pump and ASI-100 automated sample injector). Aliquots ranging between 0.2 mL - 1.5 mL of a 10 mg/mL solution were injected. A 5-40% gradient of acetonitrile in water (both containing 0.1% TFA) was used over 38.5 min followed by a step increase to 80% acetonitrile for 2 min, and a reduction to 5% for final 5 min. Fractions containing the desired product were collected and freeze dried, yielding the pure products as the TFA salt.

l-Ala-d-Glu (AE)

| Compound | mass/volume *per* coupling | mmol *per* coupling |
| --- | --- | --- |
| Fmoc-L-Ala-OH | 49.8 mg | 0.16 |
| HBTU | 60.7 mg | 0.16 |
| DIPEA | 55.7 µL | 0.32 |

The peptide was synthesised according to the above general procedures on Fmoc-D-Glu-(O*t*Bu) Wang resin (50 mg, 0.032 mmol; resin loading 0.64 mmol g^-1^). Purification by HPLC yielded the title compound as a fluffy white solid (4.5 mg, 13.6 µmol, 42%). ^1^H NMR (600 MHz, D_2_O) 1.47 (3H, d, *J* 7.1 Hz, Ala βCH_3_), 1.95 (1H, m, Glu βH_A_H_B_), 2.13-2.21 (1H, m, Glu βCH_A_H_B_), 2.42 (2H, t, *J* 7.3 Hz, Glu γCH_2_), 4.04 (1H, q, *J* 7.1 Hz, Ala αCH), 4.38 (1H, m, Glu αCH); ^13^C NMR (125 MHz, D_2_O) 17.2 (Ala βCH_3_), 26.4 (Glu βCH_2_), 30.7 (γCH_2_), 49.7 (Ala αCH), 53.1 (Glu αCH), 171.5 (Ala αCO), 175.8 (Glu αCO), 177.7 (Glu γCO); *m/z* (ES+) [M+H]^+^ 219.5, [2M+H]^+^ 437.5, [3M+H]^+^ 655.8.

l-Ala-γ-d-Glu-l-Lys (AEK)

| Compound | mass/volume *per* coupling | mmol *per* coupling |
| --- | --- | --- |
| Fmoc-D-Glu-O*t*Bu | 60.6 mg | 0.14 |
| Fmoc-L-Ala-OH | 44.4 mg | 0.14 |
| HBTU | 54.0 mg | 0.14 |
| DIPEA | 49.6 µL | 0.29 |

The peptide was synthesised according to the above general procedures, using Fmoc-Lys(Boc) Wang resin (50 mg, 0.029 mmol; resin loading 0.57 mmol g^-1^). Purification by HPLC yielded the title compound as a fluffy white solid (6.3 mg, 11.0 µmol, 38%). ^1^H NMR (600 MHz, D_2_O) 1.33-1.44 (2H, m, Lys γCH_2_), 1.47 (3H, d, *J* 7.1, Ala βCH_3_), 1.58-1.64 (2H, m, Lys δCH_2_), 1.66-1.73 (1H, m, Lys βCH_A_H_B_), 1.78-1.86 (1H, m, Lys βCH_A_H_B_), 1.93-2.00 (1H, m, Glu βH_A_H_B_), 2.13-2.18 (1H, m, Glu βH_A_H_B_), 2.31-2.39 (2H, m, Glu γCH_2_), 2.91 (2H, t, *J* 7.6, Lys εCH_2_), 4.04 (1H, q, *J* 7.1, Ala αCH), 4.27 (1H, dd, *J* 9.1, 5.1, Lys αCH), 4.31 (1H, dd, *J* 8.8, 5.3, Glu αCH); ^13^C NMR (125 MHz, D_2_O) 17.2 (Ala βCH_3_), 22.7 (Lys γCH_2_), 26.8 (Lys δCH_2_), 27.1 (Glu βCH_2_), 30.6 (Lys βCH_2_), 32.0 (Glu γCH_2_), 39.8 (Lys εCH_2_), 49.7 (Ala αCH), 53.1 (Glu αCH), 53.3 (Lys αCH), 171.5 (Ala αCO), 175.6 (Glu γCO), 175.7 (Glu αCO), 176.6 (Lys αCO); *m/z* (ES+) [M+H]^+^ 347.6, [2M+H]^+^ 693.8.

l-Ala-γ-d-Glu-Lan (AELan)

Firstly, (Teoc, TMSE/Fmoc) D,L-lanthionine was prepared as previously reported,^48^ and subsequently modified as detailed below to allow the synthesis of the desired γ-Glu linkage by SPPS.

2-(trimethylsilyl)ethyl (12*R*)-12-((((9H-fluoren-9-yl)methoxy)carbonyl)amino)-2,2-dimethyl-6-oxo-8-((2-(trimethylsilyl)ethoxy)carbonyl)-5-oxa-10-thia-7-aza-2-silatridecan-13-oate (**2**)

A solution of lanthionine **1** (65 mg, 0.10 mmol) and 4-(dimethylamino)pyridine (1.9 mg, 0.015 mmol) in anhydrous THF (0.5 mL) was cooled to 0 °C under Ar before adding 2-(trimethylsilyl)ethanol (29 µL, 0.20 mmol). To this, DCC (27 mg, 0.13 mmol) was added and the solution was warmed to rt. After 24 h, the solution was filtered and the residue washed with EtOAc before removal of the solvent under reduced pressure. The residue was redissolved in EtOAc (20 mL) and washed with aqueous NaHCO_3_ (2 x 15 mL) and brine (20 mL), then dried (MgSO_4_ and the solvent removed under reduced pressure. Purification by flash column chromatography (pet. ether:EtOAC, 9:1 → 6:1) yielded lanthionine **2** (32 mg, 0.04 mmol, 40%) as a 1:1 mix of two diastereomers. R_f_ 0.24 (pet. ether:EtOAC, 5:1); ^1^H NMR (700 MHz, CDCl_3_) 0.03-0.07 (27H, m, Si(CH_3_)_3_), 0.97-1.06 (6H, m, CH_2_CH_2_Si), 2.97-3.08 (4H, m, CHCH_2_S), 4.14-4.32 (7H, m, OCH_2_CHAr, CH_2_CH_2_Si), 4.39-4.42 (2H, m, OCH_2_CHAr), 4.54-4.59 (2H, m, CHCH_2_S), 5.51 (1H, br, NHFmoc), 5.72 (1H, d, *J* 7.3, NHTeoc diastereomer 1), 5.81 (1H, d, *J* 6.6, NHTeoc diastereomer 2), 7.33 (2H, t, *J* 7.4, ArH(3)), 7.41 (2H, t, *J* 7.4, ArH(4)), 7.62-7.65 (2H, m, ArH(2)), 7.78 (2H, d, *J* 7.5, ArH(5)); ^13^C NMR (175 MHz, CDCl_3_) -1.30 (Si(CH_3_)_3_), 17.6 (CH_2_CH_2_Si), 17.8 (CH_2_CH_2_Si), 35.5 (CHCH_2_S), 35.6 (CHCH_2_S), 35.8 (CHCH_2_S), 36.0 (CHCH_2_S), 47.2 (OCH_2_CHAr), 53.9 (CHCH_2_S), 54.0 (CHCH_2_S), 54.1 (CHCH_2_S), 63.7 (CH_2_CH_2_Si), 64.5 (CH_2_CH_2_Si), 64.6 (CH_2_CH_2_Si), 67.4 (OCH_2_CHAr), 120.1 (C5), 125.2 (C2), 125.3 (C2), 127.1 (C3), 127.8 (C4), 141.4 (C6), 143.9 (C1), 155.9 (NHCO_2_Fmoc), 156.3 (NHCO_2_Teoc), 170.6 (CO_2_TMSE), 170.8 (CO_2_TMSE); *m/z* (HRMS, ES+) predicted [C_37_H_58_N_2_O_8_SSi_3_+Na]^+^ 797.3114, found [C_37_H_58_N_2_O_8_SSi_3_+Na]^+^ 797.3117.

2-(trimethylsilyl)ethyl (12*R*)-12-amino-2,2-dimethyl-6-oxo-8-((2-(trimethylsilyl)ethoxy)carbonyl)-5-oxa-10-thia-7-aza-2-silatridecan-13-oate (**3**)

To a solution of lanthionine **2** in MeCN (800 µL) under Ar, diethylamine (200 µL) was added. The reaction was stirred for 30 min at rt before removal of the solvent under reduced pressure. Purification by flash column chromatography (pet. ether:EtOAC, 5:1 → 1:1) yielded lanthionine **3** (9.5 mg, 0.02 mmol, 44%) as a 1:1 mix of two diastereomers. R_f_ 0.74 (pet. ether:EtOAC, 1:1); ^1^H NMR (700 MHz, CDCl_3_) 0.03-0.05 (27H, m, Si(CH_3_)_3_), 0.98-1.03 (6H, m, CH_2_CH_2_Si), 1.73 (2H, br s, NH_2_), 2.76-2.79 (1H, m, CHCH_A_H_B_S (3) diastereomer 1), 2.83-2.86 (1H, m, CHCH_A_H_B_S (3) diastereomer 2), 2.92-3.00 (2H, m, CHCH_A_H_B_S (3) diastereomer 1 and CHCH_A_H_B_S (3) diastereomer 2), 3.01-3.03 (2H, m, CHCH_2_S (2)), 3.59-3.63 (1H, m, CHCH_2_S (4)), 4.15-4.28 (6H, m, CH_2_CH_2_Si), 4.55-4.58 (1H, m, CHCH_2_S (1)), 5.84 (1H, d, *J* 7.4, NHTeoc diastereomer 1), 6.08 (0.5H, d, *J* 7.7, NHTeoc diastereomer 2); ^13^C NMR (175 MHz, CDCl_3_) -1.30 (Si(CH_3_)_3_), 17.6 (CH_2_CH_2_Si), 17.8 (CH_2_CH_2_Si), 35.6 (CHCH_2_S), 35.7 (CHCH_2_S), 38.0 (CHCH_2_S), 38.1 (CHCH_2_S), 54.1 (CHCH_2_S), 54.2 (CHCH_2_S), 54.5 (CHCH_2_S), 54.8 (CHCH_2_S), 63.6 (CH_2_CH_2_Si), 63.9 (CH_2_CH_2_Si), 64.4 (CH_2_CH_2_Si), 156.4 (NHCO), 170.9 (CO_2_TMSE), 173.9 (CO_2_TMSE); *m/z* (HRMS, ES+) predicted [C_22_H_48_N_2_O_6_SSi_3_+H]^+^ 553.2614, found [C_22_H_48_N_2_O_6_SSi_3_+H]^+^ 553.2620.

Following preparation of lanthionine **3**, the tripeptide was synthesised by SPPS as detailed below. Protocols for Fmoc deprotection, Ala coupling and cleavage are as described in the general procedures.

To prepare the required resin, the symmetrical anhydride of Fmoc-D-Glu(OAll)-OH was synthesised. Fmoc-D-Glu(OAll)-OH (0.90 g, 2.20 mmol) was dissolved in CH_2_Cl_2_ under Ar, and cooled to 0 °C. DIC (0.5 eq, 170 µL, 1.10 mmol) was added, and the solution stirred at 0 °C for 30 min, before removing the solvent *in vacuo*. The residue was redissolved in DMF (2 mL) and added to pre-swollen Wang resin (400 mg, 0.176 mmol; loading 0.44 mmol g^-1^), followed by a solution of DMAP (2.15 mg, 0.0176 mmol) in DMF (1 mL). After 8 h, the solution was removed and the resin was washed with CH_2_Cl_2_ (3 x 2 mL) and DMF (3 x 2 mL). The resin loading was confirmed to be complete using an Fmoc loading test.

| Compound | mass/volume *per* coupling | mmol *per* coupling |
| --- | --- | --- |
| Fmoc-L-Ala-OH | 274 mg | 0.88 |
| HBTU | 334 mg | 0.88 |
| DIPEA | 307 µL | 1.76 |

After preparation of the resin, the Fmoc group was removed and the Ala residue added. The resin was washed with DMF (2 x 2 mL), CH_2_Cl_2_ (3 x 2 mL), MeOH (3 x 2 mL) and diethyl ether (3 x 2 mL), and dried under vacuum overnight.

A portion of the dried resin was removed (21 mg, 9.05 µmol) and swollen in DMF. To remove the Allyl group, Pd(PPh_3_)_4_ (2 eq, 21 mg, 0.0181 mmol) and PhSiH_3_ (10 eq, 11.2 µL, 0.0905 mmol) were first dissolved in DMF:CH_2_Cl_2_ (1:1 v/v, 1 mL). This solution was added to the resin and the solution stirred in the dark for 2 h. The deprotection solution was then removed, and the resin washed with CH_2_Cl_2_ (5 x 2 mL), 0.5% sodium diethyldithiocarbamate in DMF (5 x 3 mL) and DMF (5 x 2 mL).

To add the Lan residue, lanthionine **3** (2 eq, 9.5 mg, 0.0181mmol), PyAOP (5 eq, 26.6 mg, 0.0453 mmol) and HOAt (5 eq, 6.2 mg, 0.0453 mmol) were added to a glass vial, followed by DIPEA (10 eq, 15.8 µL, 0.0905 mmol). This solution was left at rt for 2 min before addition to the resin. The resin was shaken at rt for 2 h before removal of the coupling solution and washing the resin with DMF (4 x 2 mL). The silyl protecting groups were removed from the peptide by treating the resin with a solution of TBAF (1 M in THF, 1 mL) in DMF (1 mL). After 1 h the solution was removed and the resin washed with DMF (6 x 2 mL). The final Fmoc group was then removed and the peptide cleaved from the resin. Purification by HPLC yielded the title compound as a fluffy white solid, as a 1:1 mix of diastereomers (4.0 mg, 6.28 µmol, 68%). ^1^H NMR (600 MHz, D_2_O) 1.53-1.54 (3H, m, Ala βCH_3_), 2.00-2.05 (1H, m, Glu βH_A_H_B_), 2.18-2.25 (1H, m, Glu βH_A_H_B_), 2.34-2.37 (2H, m, Glu γCH_2_ diastereomer 1), 2.40-2.44 (2H, m, Glu γCH_2_ diastereomer 2), 2.93-2.98 (1H, m, Lan βH_A_H_B_ (3) diastereomer 2), 3.05-3.10 (1H, m, Lan βH_A_H_B_ (2) diastereomer 2), 3.11-3.14 (2H, m, Lan βH_A_H_B_ (2) and (3) diastereomer 2), 3.33-3.36 (1H, m, Lan βH_A_H_B_ (3) diastereomer 1), 3.41-3.44 (1H, m, Lan βH_A_H_B_ (3) diastereomer 1), 3.61-3.64 (1H, m, Lan βH_A_H_B_ (2) diastereomer 1), 3.73-3.76 (1H, m, 1H, m, Lan βH_A_H_B_ (2) diastereomer 1), 3.81-3.82 (1H, m, Lan αCH (1) diastereomer 1), 3.97-3.98 (1H, m, Lan αCH (1) diastereomer 2), 4.09-4.13 (1H, m, Ala αCH), 4.33-4.39 (1H, m, Glu αCH), 4.55-4.56 (1H, m, Lan αCH (4) diastereomer 2), 4.77 (1H, m, Lan αCH (4) diastereomer 1); *m/z* (ES+) [M+H]^+^ 409.6, (ES-) [M-H] ^-^ 407.5.
